# Editorial: Saliva and Oral Microbiota: From Physiology to Diagnostic and Therapeutic Implications

**DOI:** 10.3389/fphys.2020.637599

**Published:** 2021-01-13

**Authors:** Andrea Santarelli, David T. W. Wong, Lorenzo Lo Muzio

**Affiliations:** ^1^Department of Clinic Specialistic and Stomatological Sciences, Marche Polytechnic University, Ancona, Italy; ^2^School of Dentistry, University of California, Los Angeles, Los Angeles, CA, United States; ^3^Department of Clinical and Experimental Medicine, University of Foggia, Foggia, Italy

**Keywords:** saliva, oral microbiota, omics sciences, point-of-care diagnosis, dysbiosis

Saliva is a remarkably complex fluid with many properties and functions which are essential for both oral and general health. In the last years, technological advancements, especially in “-omics” studies and bioinformatics tools, recognized saliva as a pool of biological markers. Saliva represents a safe and non-invasive source of potentially useful information that could help to evaluate the state of health and the presence of disease (Dawes and Wong, 2019). Furthermore, the new developments in the field of cancer biology suggest that the so called “salivaomics” is a promising approach for early detection of cancer, first and foremost the oral cancer. However, the importance of the “classic” function of saliva is attested by the important consequences on quality of life suffered by subject with hyposalivation and xerostomia and by the efforts made in strategies for restoring salivary gland function in patients with parenchyma and ducts anomalies (Togni et al.). Moreover, the demographic transition in western countries is leading to the progressive aging of the population, increasing the number of elderlies that may experience occlusal and masticatory problems that in turns may imbalance water transportation in salivary gland as seen in the animal model (Saito et al.).

The extensive investigations of saliva highlight the specific characteristics of oral microenvironment with its bacterial population and its relationship with the whole body. In the last years, there has been a growing interest in understanding oral microbiota and its relationship with the oral health status and with local and systemic diseases. The study of oral microbial ecology represents another emerging connection between oral and systemic health, opening up the possibility for new diagnostic and therapeutic perspectives. Oral microbiota represents a heterogeneous group of microbial species colonizing all the oral cavity surfaces. About 700 bacterial species have been identified in oral cavity with 35% of them have not been cultured. To determine bacterial ecosystem, high-throughput sequencing methods have been developed, representing a leap toward characterization of oral microbial community and their host interaction (He et al., 2018). Recently, results from studies based on genome sequencing have started shedding light on the existence of site-specific microbial patterns that might be considered as “healthy oral microbiota.” The main obstacle to this definition is the highly variable composition of microbial communities. Nevertheless, ecological imbalance of microbial community, called dysbiosis, has been extensively studied both in human and animal models. It is characterized by the loss of beneficial microbes, expansion of pathogenic microbes, and general loss of microbial diversity.

An increasing number of studies have shown that oral microbiota plays a role in the development of oral diseases, such as dental caries, periodontal disease, and oral stomatitis. The salivary microbial species could help to discriminate patients with treated, well-maintained chronic periodontitis from healthy controls with similar gingival inflammation levels. Several species, such as *Treponema* spp., *Prevotella* spp., and *Capnocytophaga* spp., have been significantly associated with treated and well-maintained periodontitis. While *Leptotrichia buccalis, Corynebacterium matruchotii, Leptotrichia hofstadii*, and *Streptococcus intermedius*, resulted significant indicators of healthy periodontal condition (Acharya et al.). Moreover, the inoculation of *Porphyromonas gingivalis* in a *in vivo* mouse model showed the developmental of the periodontal disease, resulting in oral microbiome dysbiosis (Walkenhorst et al.).

Lately, is becoming evident that oral dysbiosis is associated with head and neck cancer development and with the pathogenesis of systemic diseases. Furthermore, it also seems to be involved in gastrointestinal neoplasms, especially esophageal, gastric, pancreatic, and colorectal cancers, paving the way for developing specific oral microbiota test to allow early cancer detection (Mascitti et al.). Liquid biopsy represents a non-invasive method for the detection of diagnostic and prognostic biomarkers in body fluids of oncologic patients. The salivary liquid biopsy provides several promising clinical uses in cancer management: it is painless, accessible, and low cost and represents a very helpful source of diagnostic and prognostic biomarker detection. Even if standardized protocols for isolation, characterization, and evaluation are needed, recent data suggest that saliva may be successfully included in future clinical diagnostic processes, with a considerable impact on early treatment strategies and a favorable outcome (Cristaldi et al.).

Regarding systemic diseases, the *Streptococcus oralis* has been recognized as a pathogen to cause multiorgan failure by contributing to the formation of microthrombus. Mice infected by Streptococcus oralis showed a downregulation of hepatic BMAL1 gene expression, a downregulation of the coagulation factor VII and an upregulation of the coagulation factor XII *in vitro* and *in vivo*. Thus, these results could improve the treatment of hepatic sepsis by the regulation of the BMAL1 gene expression (Chen et al.).

Oral microbial dysbiosis is known to increase susceptibility of an individual to develop the rheumatoid arthritis. It has been observed a characteristic compositional change of salivary microbes in individuals at high-risk for rheumatoid arthritis, suggesting oral microbiota dysbiosis occurs in the pre-clinical stage of this condition (Tong et al.). Moreover, the microbiota has been observed to have a growing relationship with diabetes. Microbiota imbalance has been hypothesized to be involved in the regulation of energy metabolism and the inflammatory immune response. In a type 2 diabetes mouse model, the insulin resistance was improved, and the pancreatic markers of inflammation were reduced after Fecal Microbiota Transplantation, providing a novel potential treatment strategy (Wang et al.).

Finally, studies pointed out that antibacterial toothpaste (Sparabombe et al.), all-natural polyherbal mouthwash (Shang et al.) and antibacterial mouthwashes could lead to an alteration of oral microbiota and of oral microbiota salivary nitrate, a substrate of oral and intestinal microbiota, metabolism. The consequences on the circulating Nitric Oxide levels are still completely unclear (Zhurakivska et al.). However, the alterations of oral microbiota could lead to modifications in plasmatic Nitric Oxide content, resulting in increases in systolic blood pressure. Moreover, the tongue cleaning frequency resulted as a predictor of chlorhexidine-induced changes in systolic blood pressure and tongue microbiome composition. These data suggest that management of the tongue microbiome together with adequate dietary intake of nitrate provide an opportunity for the improvement of resting systolic blood pressure (Tribble et al.).

In conclusion, this Research Topic has aimed to provide a complete and comprehensive overview of saliva, passing through oral microbiota interactions, and their role in health and disease, describing also point-of-care diagnostic and therapeutic possibilities.

## Author Contributions

All authors listed have made a substantial, direct and intellectual contribution to the work, and approved it for publication.

## Conflict of Interest

The authors declare that the research was conducted in the absence of any commercial or financial relationships that could be construed as a potential conflict of interest.
